# Effect of HIV and malaria parasites co-infection on immune-hematological profiles among patients attending anti-retroviral treatment (ART) clinic in Infectious Disease Hospital Kano, Nigeria

**DOI:** 10.1371/journal.pone.0174233

**Published:** 2017-03-27

**Authors:** Feyisayo Ebenezer Jegede, Tinuade Ibijoke Oyeyi, Surajudeen Abiola Abdulrahman, Henry Akwen. Mbah, Titilope Badru, Chinedu Agbakwuru, Oluwasanmi Adedokun

**Affiliations:** 1 Family Health International-360 Plot 1073-A1 GODAB Plaza, Area 3 Garki-Abuja, Nigeria; 2 Biological Science Department, Bayero University, Kano, Nigeria; 3 Department of Public Health, Penang Medical College, George Town, Penang, Malaysia; 4 LabTrail, Global LLC, Smyrna DE, United States of America; Universidade Nova de Lisboa Instituto de Higiene e Medicina Tropical, PORTUGAL

## Abstract

**Background:**

Human immunodeficiency virus (HIV) and malaria co-infection may present worse health outcomes in the tropics. Information on HIV/malaria co-infection effect on immune-hematological profiles is critical for patient care and there is a paucity of such data in Nigeria.

**Objective:**

To evaluate immune-hematological profiles among HIV infected patients compared to HIV/malaria co-infected for ART management improvement.

**Methods:**

This was a cross sectional study conducted at Infectious Disease Hospital, Kano. A total of 761 consenting adults attending ART clinic were randomly selected and recruited between June and December 2015. Participants’ characteristics and clinical details including two previous CD4 counts were collected. Venous blood sample (4ml) was collected in EDTA tube for malaria parasite diagnosis by rapid test and confirmed with microscopy. Hematological profiles were analyzed by Sysmex XP-300 and CD4 count by Cyflow cytometry. Data was analyzed with SPSS 22.0 using Chi-Square test for association between HIV/malaria parasites co-infection with age groups, gender, ART, cotrimoxazole and usage of treated bed nets. Mean hematological profiles by HIV/malaria co-infection and HIV only were compared using independent t-test and mean CD4 count tested by mixed design repeated measures ANOVA. Statistical significant difference at probability of <0.05 was considered for all variables.

**Results:**

Of the 761 HIV infected, 64% were females, with a mean age of ± (SD) 37.30 (10.4) years. Prevalence of HIV/malaria co-infection was 27.7% with *Plasmodium falciparum* specie accounting for 99.1%. No statistical significant difference was observed between HIV/malaria co-infection in association to age (p = 0.498) and gender (p = 0.789). A significantly (p = 0.026) higher prevalence (35.2%) of co-infection was observed among non-ART patients compared to (26%) ART patients. Prevalence of co-infection was significantly lower (20.0%) among cotrimoxazole users compared to those not on cotrimoxazole (37%). The same significantly lower co-infection prevalence (22.5%) was observed among treated bed net users compared to those not using treated bed nets (42.9%) (p = 0.001). Out of 16 hematology profiles evaluated, six showed significant difference between the two groups (i) packed cell volume (p = <0.001), (ii) mean cell volume (p = 0.005), (iii) mean cell hemoglobin concentration (p = 0.011), (iv) absolute lymphocyte count (p = 0.022), (v) neutrophil percentage count (p = 0.020) and (vi) platelets distribution width (p = <0.001). Current mean CD4 count cell/μl (349±12) was significantly higher in HIV infected only compared to co-infected (306±17), (p = 0.035). A significantly lower mean CD4 count (234.6 ± 6.9) was observed among respondents on ART compared to non-ART (372.5 ± 13.2), p<0.001, mean difference = -137.9).

**Conclusion:**

The study revealed a high burden of HIV and malaria co-infection among the studied population. Co-infection was significantly lower among patients who use treated bed nets as well as cotrimoxazole chemotherapy and ART. Six hematological indices differed significantly between the two groups. Malaria and HIV co-infection significantly reduces CD4 count. In general, to achieve better management of all HIV patients in this setting, diagnosing malaria, prompt antiretroviral therapy, monitoring CD4 and some hematology indices on regular basis is critical.

## Introduction

Available research findings suggests that both human immunodeficiency virus (HIV) and malaria parasite infection act synergistically resulting in worse health outcomes [1]. Anemia, increase plasma viral load and decrease CD4 count among HIV infected are some of the worse health outcomes due to frequent episodes of symptomatic malaria [2] [3][4]. HIV may facilitates geographic expansion of malaria in areas where HIV prevalence is high. Therefore repeated increase in HIV viral load due to recurrent co-infection may be an important factor promoting the spread in sub-Sahara Africa [5].

In tropical countries pathophysiological, clinical and epidemiological interactions between HIV and pathogenic organisms especially malaria parasites constitute a concern of public health implication [6]. Opportunistic infections caused by viruses, parasites, bacteria, fungi and other pathogens remain as major causes of mortality among HIV patients [7]. Daily cotrimoxazole chemotherapy is recommended by the world health organization (WHO) as a major strategy of preventing opportunistic infections among HIV infected clients in Sub-Saharan Africa [8]. Despite strong research evidence of its strong antimalarial prophylactic properties, the efficacy and long term usage may be limited because of potential antifolate resistance drawback [9]. HIV and malaria infection particularly with plasmodium parasites are both pathogens that induce significant perturbation and activation of the immune system. Both pathogens (HIV and malaria) may be contributing factors in the modification of each other’s development, disease severity and disease progression rate [10]. The greatest burden of disease due to both HIV and malaria (predominantly *Plasmodium falciparum*) occurs commonly in Sub-Saharan Africa [11]. The potential consequences of both diseases’ interaction, including understanding their reciprocal effects on host immune response and their combined effect on host response to other pathogens is important [11].

Infection of HIV and malaria are among the two most important global health problems of developing countries including Nigeria which was reported to cause more than 4 million deaths a year, with HIV infection increasing the risk of and severity of malaria infection and burdens [12]. Furthermore, HIV in turn facilitates the rate of malaria transmission which in turn causes strong CD4 cell activation and up-regulation of pro-inflammatory and cytokines production which create an ideal microenvironment for the spread of HIV among CD4 cells for rapid HIV-1 replication [13]. HIV infection and malaria is worrisome because they co-exist mostly in African countries [5][14] but studies differs in their findings on the interactions between the two infections [15] [16]. In an environment where malaria is common, the incidence of clinical malaria episodes is reported to be higher in patients with CD4 cell counts <200 cells/μl than in those with CD4 cell counts >500 cells/μl. [17]. Hence high incidence of fever identified among immunosuppressed adults may lead to misclassification of illness episodes as malaria [17]. HIV and malaria both destroy important cells required for proper immunological and hematological functioning of the body [18]. Enhanced T-cell activation in HIV and malaria co-infected patients could worsen the immune response to both diseases [19]. Non-immune HIV-infected patients are significantly more likely to have severe malaria than non-immune non-HIV-infected patients with odds ratio, 4.15 (95% confidence interval, 1.57–10.97; p = 0.003) [20]. This suggest that HIV-infected non-immune adults are at increased risk of severe malaria and the risk is associated with a low CD4 cell counts [20].

Hematological abnormalities reported previously in HIV and malaria co-infected in Nigeria includes; anemia, thrombocytopenia, neutropenia and leucopenia which were significantly higher among HIV and malaria infected population compared to HIV mono infection [21]. Several studies have provided evidence that anemia is the most common hematological abnormality in HIV and malaria co-infected patients [4][3] [22][23] [24]. However, some studied have made contrary observations that hematological profiles among HIV and malaria co-infection are not significantly different compared to HIV mono infected [25][26]. Previous research data on HIV and malaria co-infection in Nigeria revealed wide variation in prevalence which range from 2.9% in Lagos [3] to 93.3% in Port Harcourt [21]. Data reported in many African countries on HIV and malaria co-infection and effect on immune-hematological profiles has not been comprehensively evaluated. The Hematological profiles previously evaluated in these studies were on specific and limited indices. For example, HIV and malaria co-infection and effect on Packed cell volume, hemoglobin, white blood cell count, without white blood cell differential count or combination of two or three hematological indices [4][3][27] [28] [29] [30][31].

The aim of this study was to evaluate the immune-hematological profiles in HIV infected patients compared to HIV/malaria co-infected patients to provide information to improve on ART (Antiretroviral therapy) management.

## Materials and methods

### Setting

The study was conducted at Infectious Disease Hospital (IDH) Kano, located at France road in Fagge local government area of Kano State in North West of Nigeria. It was established in the early 60s as an isolation unit for smallpox patients. Subsequently, the unit was expanded into a fully-fledged secondary level, state owned public hospital and now caters for all epidemic diseases prevalent in the State. The hospital provides services to a catchment area including Kano State, neighboring States and also to patients from the Republic of Niger. Presently, the hospital has a capacity of about 250 beds, a new multi-drug resistant (MDR) tuberculosis clinic and wards. With funds from the President’s Emergency Plan for AIDS Relief (PEPFAR) through the United States Agency for International Development (USAID), Family Health International-360 (FHI-360) has supported IDH Kano to provide comprehensive ART program since April 2005 till date. The ART laboratory provides HIV serological screening and monitoring tests such as CD4 enumeration, clinical chemistry, hematology indices as well as others like pregnancy, hepatitis, syphilis and tuberculosis. A total of 9,854 HIV positive patients have so far been placed on ART and 6,073 patients are currently on antiretroviral drugs as at August, 2016. The facility was purposefully selected because of patient load, presumed availability of patient information in both electronic management system software and patient folders. The facility is also the first secondary health facility owned by State government to commence ART services in Kano State.

### Eligibility criteria for the study

We included HIV infected male and female adult patients aged 18 years and above receiving HIV care and treatment services at IDH Kano. These patients were duly registered into the HIV care and treatment program in the hospital and had willfully consented by signing consent forms to participate in the study. We excluded pregnant HIV infected women and any HIV infected patient who has been on antimalarial drug in the past fourteen days (2 weeks) as well as all those who did not consent to participate.

### Sample size determination

Sample size was calculated using STATA software with the following assumptions for each hematological parameter:

1. Hemoglobin concentration g/dl

Assuming an effect size of 2g/dl required for hemoglobin. A standard deviation of 2.41 in similar study was used [26]. To achieve 80% power to detect this difference with a significance level of 5%, it was estimated that 23 subjects per group were required.

2. Red blood cells (RBC) count X10^12/l^

Assuming an effect size of 1.5X10^12^/l required for RBC. A standard deviation of 4.28 in similar study was used [26]. To achieve 80% power to detect this difference with a significance level of 5% it was estimated that 200 subjects per group were required.

3. Platelets count X10^9/l^

Assuming an effect size of 15X10^9^/l required for Platelets count. A standard deviation of 51.9 in similar study was used [21]. To achieve 80% power to detect this difference with a significance level of 5% it was estimated that 188 subjects per group were required.

RBC yielded the largest sample size. To account for 5% non-response rate, a total of 420 HIV infected patients (210 HIV/malaria co-infected and 210 HIV infected patients) minimum was required for this study but a total of 761 patients were actually enrolled for the study.

### Study design

This was a cross sectional study involving 761, randomly selected consenting HIV infected adults attending HIV treatment and care clinic of IDH Kano.

### Ethical consideration

The protocol was reviewed by Kano State Hospitals Management Board local ethical committee and written approval was provided (REF: HMB/GEN/488/1 of 17th April 2015). Subsequently, respondents in the study population voluntarily singed consent forms before enrollment into the study.

### Data collection and sampling technique

We used a simple random sampling technique to select consenting participants from a sampling frame that included all HIV positive patients enrolled into care between June to December 2015. We used a detailed anonymous pre-tested questionnaire to capture participants’ basic characteristics and clinical information with the help of trained healthcare workers. These information included: clients’ code, age, sex, occupation, educational status, use of cotrimoxazole, socio-economic characteristics, as well as history of current use of anti-malaria and use of insecticide treated mosquito nets. Other relevant clinical information retrieved included ART therapy regimen, World Health Organization (WHO) HIV clinical stage. In addition, two CD4 count measurements (firstly at baseline, secondly at follow–up) from 2005 to 2015 were retrieved from patients folders or Lafiaya Information management system (LAMIS) software with the assistance of trained data entry clerks.The most current CD4 count which is the third measurements was determined during this study. All information were entered into excel files, reviewed for correctness, completeness and consistency. We ensured data confidentiality through the use of patient coding system and restricting access to the data.

### Blood sample collection

Blood samples were collected from consenting HIV infected patients daily from Monday to Friday in the morning before 11:30 am until the required sample size was reached. For each participant, 4ml of venous blood was aseptically collected using sterile vacutainer needle/holder into ethylenediametetracaetic acid (EDTA) tube. The samples were mixed properly to avoid blood clot before laboratory investigations.

### Malaria parasite diagnosis

To screen for malaria parasites, we used malaria rapid diagnostic test (RDT) kits as described by the manufacturer’s (Standard Diagnostics Boline, 2013). Briefly, all test devices were allowed to stand at room temperature for 15 minutes. Twenty microliters (20μl) of EDTA whole blood sample was added to sample test device, followed by 3 drops of cleaning buffer. The reaction was allowed to stand at room temperature for 15 minutes. The appearance of distinct red line at the control and test region was observed for result interpretation. Two distinct red lines indicate positive malaria antibody of *Plasmodium falciparum or Plasmodium vivax*. For test validation, a distinct red line was required in the control region. Any contrary result was attributed to a non-functional test kit or human error. In such cases a repeat of the procedure with another testing cartridge of standard diagnostics test kit was carried out.

Regardless of rapid diagnostic test (RDT) results, blood smear microscopy method was used for malaria parasite confirmation. Using the same clean microscope slide, about 6μl of EDTA whole blood was used for thick smear and 3μl for thin smear preparation.The smears were allowed to air dry and the thin smear was fixed in absolute methanol before the slide was stained with freshly prepared 3% Giemsa stain for 45 minutes. The preparation was differentiated with 7.2 pH buffer distilled water, washed and air dried before it was examined using light microscopy. The procedure for both thick and thin blood smear microscopy was adopted using 3% Giemsa staining procedures as described by World Health Organization (WHO) [32].

### Complete blood count (full automation)

Complete blood count and platelets analysis were carried out using automated Sysmex XP-300 analyzer with strict adherence to the manufacturer instruction. Daily quality control was carried out to validate each test run as described by equipment manufacturer [33].

### CD4 estimation—Partec cyflow technique

We put 20μl of CD4+- PE monoclonal antibody in labelled Partec (Rohren) tubes and added 20μl of well mixed EDTA blood. This content was mixed together several times for 2 minutes and incubated in the dark for 15 minutes at room temperature with intermittent mixing every 5 minutes. After incubation, 800μl of CD4 diluting buffer was added to each preparation, mixed properly before analyzed on the Cyflow counter as describe by equipment manufacturer [34].

### Statistical analysis

Data generated were entered into excel file, reviewed cleaned and imported into Statistical Package for Social Sciences (SPSS) software version 22.0 for analysis. Univariate analysis which includes descriptive statistics like frequencies and exploration of the distribution of all variables of interest was performed. Association between categorical variables was tested using chi-square test i.e. association between HIV and malaria co-infection status in relation to age group, gender, cotrimoxazole, chemotherapy, treated beds net and ART status. The mean of hematological parameters by HIV and malaria co-infection and HIV only status was generated and compared using independent sample t-test. Repeated measures of analysis of variance (ANOVA) test was used to compare the mean CD4 count measurement at baseline, follow-up and current CD4 count respectively. All tests were two-tailed with statistical significance set at 5% (0.05).

## Results

### Basic characteristics and clinical information of study population

Of the 761 HIV positive subjects enrolled in this study, 487 (64%) were females and 274 (34%) males. The mean age± (SD) was 37.30 ±10.4 years, with a range of 18–70 years. Majority (73.3%) were Hausa/Fulani by tribe and about 85% resided in urban settings. Majority (65%) had some level of education from primary to tertiary level. About three quarters (75.8%) of the participants did not complained about their health at the time of visit during this study. Of those that complained about their health, 43.5% presented with fever and headache/others while the rest complained of body pain or body weakness/others combined. Approximately three out of every four (74.2%) respondents use insecticide treated mosquito bed nets (Table 1).

**Table 1:** Baseline Characteristics of HIV Patients Attending ART Clinic at Infectious Disease Hospital Kano Between June to December 2015 (n = 761).

Variable	Number (%)	Variable	- Number (%)
**Gender**		**Ethnicity**	
Male	274 (36)	Hausa/Fulani	558 (73.3)
Female	487 (64)	Yoruba	21 (2.8)
**Age Group (years)**		Igbo	42 (5.5)
Less than 30	161 (21.15)	Others	140 (18.4)
30–39	292 (38.4)	**Complaint reported**	
40–49	197 (25.9)	No	577 (73.3)
50–59	85 (11.2)	Yes	184 (24.2)
60 Above	26 (3.4)	**Type of complaint**	
**Place of Resident**		Fever/Headache/others	80 (43.5)
Rural	116 (15.2)	Body pain/Weakness/others	50 (27.2)
Urban	645 (84.8)	Stomach Upset/others	13 (7.1)
**Marital status**		Body rashes/Chest pain/stooling	11 (5.9)
Married	438 (57.6)	Vomiting /Others	30 (16.3)
Single	102 (13.4)		
Divorced	56 (7.4)	**Treated bed nets Usage**	
Widow/Widower	162 (21.3)	No	196 (25.76)
**Educational Status**		Yes	565 (74.24)
No Formal Education	268 (35.2)	Frequency of net Usage/week	
Some Primary Education	59 (7.8)	Frequency 1–2 days /week	15 (2.7)
Completed Primary	124 (16.3)	Frequency 3–4 days /week	84 (14.9)
Completed Secondary	232 (30.5)	Frequency 5–6 days /week	55 (9.4)
Completed tertiary	78 (10.2)	Frequency 7 days /week	411 (72.7)
		**Occupation**	
		Civil servant	85 (11.2)
		Farmer	15 (2.0)
		Business	345 (46.5)
		Artisan	16 (2.1)
		Student	29 (3.8)
		House Wife	84 (11.0)
		Un-employed	115 (15.1)
		Others	63 (8.3)

Data on clinical information showed that majority (80.9%) of the respondents were currently on ART. More than half (53.9%) of the subjects were placed on Zidovudine/Lamuvidine/Nevirapine (AZT/3TC/NVP) regimen while about 45% were on Tenofovir/Lamuvidine/Nevirapine (TDF/3TC/NVP) regimen. Based on current WHO HIV clinical staging [8], it was observed that more than half (53.9%) of the respondents were in stage II and stage III combined while stage IV accounted for over 22.1% with stage I being the least (3.7%) among HIV positive subjects. More than half (55.1%) of the participants were on daily dose of cotrimoxazole chemotherapy for prevention against opportunistic infection (Table 2).

**Table 2:** Clinical Information of HIV Patients Attending ART Clinic at Infectious Disease Hospital (n = 761).

Variables	Number (%)
**ART status**	
ART	616 (80.9)
Non-ART	145 (19.1)
**ART start Year**	
2005	52 (8.4)
2006	30 (4.9)
2007	46 (7.5)
2008	52 (8.4)
2009	46 (7.5)
2010	23 (3.7)
2011	31 (5.0)
2012	48 (7.8)
2013	47 (7.6)
2014	72 (11.9)
2015	169 (27.4)
**ART Regimen**	
TDZ/3TC/EFV	275 (44.6)
ZDV/3TC/NVP	332 (53.9)
Others	9 (1.5)
**Current WHO stage**	
Stage I	28 (3.7)
Stage II	410 (53.9)
Stage II	155 (20.4)
Stage IV	168 (22.1)
**Daily dose cotrimoxazole**	
NO	342 (44.9)
Yes	419 (55.1)

AZT,Zidovudine; NVP, Nevirapine; 3TC, Lamuvindine; TEN-,Tenofovir.

ART, Antiretroviral therapy; WHO, World health organization; %, Percentage.

Note: 27 patient data were excluded in the analysis due to missing information.

### Prevalence of HIV and malaria parasite co-infection

Prevalence of HIV and malaria was 27.7%. *Plasmodium falciparum* species infection was predominant, accounting for 99.1% of the co-infection. Only two patients (0.9%) had mixed infection of *Plasmodium falciparum* and *Plasmodium vivax*. The result of the rapid diagnostic test was lower (25.5%) compared to the gold standard blood smear microscopy method (27.7%). There was no significant difference in prevalence between gender. Participants in age group 50–59 had the highest (34.1%) prevalence closely followed (30.8%) by 60 years and above, and the least (24%) was among age group 40–49. The other age groups showed similar prevalence of about 28% but no significant difference between age groups (Table 3). Comparative analysis of prevalence based on ART status showed significantly (p = 0.026) higher prevalence of HIV and malaria co-infection among respondents who were non- ART (35.2%) compared to those on ART (26%). We found a significantly (p = 0.001) higher prevalence of malaria co-infection among respondents who were not on cotrimoxazole (37.1%) compared to those on cotrimoxazole (20.0%). Similarly, respondents who used treated bed nets had a significantly (p = 0.001) lower prevalence (22.5%) of malaria co-infection compared to those who did not use treated bed nets (42.9%) (Table 3).

**Table 3:** Prevalence of HIV and Malaria Co-infection in Relation to Age, Gender. Cotrimoxazole and Treated bed net usage among HIV Patients Attending ART Clinic at Infectious Disease Hospital Kano Between June to December 2015 (n = 761).

Variable	Number Positive (%) n = 211	Number Negative (%) n = 550	P-value*
**Prevalence HIV and Malaria (n = 761)**	211 (27.7)	550 (72.3)	-
BSM MP specie *Pf* (n = 211)	209 (99.1)	-	-
BSM MP specie *Pf&Pv* (n = 211)	2 (0.9)		
RDT MP only (n = 761)	194 (25.5)	567 (74.5)	
RDT MP specie *Pf* (n = 194)	192 (99)	-	-
RDT MP specie *Pf&Pv* (n = 194)	2 (1)		
**Age group (Years)**			
Less than 30	46 (28.6)	115 (71.4)	**0.496**
30–39	81 (27.7)	211 (72.3)	
40–49	47 (23.9)	150 (76.1)	
50–59	29 (34.1)	56 (65.9)	
60 and above	8 (30.8)	18 (69.2)	
**Gender prevalence**			
Male (n = 274)	74 (27)	200 (73)	**0.739**
Female (n = 487)	137 (28.1)	350 (71.9)	
Total	211 (27.7)	550 (72.7)	
**ART Status**			
ART	160 (26)	456 (74)	**0.026**
Non-ART	51 (35.2)	94 (64.8)	
**Co-trimoxazole use**			
No	127 (37.1)	215 (62.9)	0.001
Yes	84 (20.0)	335 (80.0)	
Treated bednet usage			
No	84 (42.9)	112 (57.1)	0.001
Yes	127 (22.5)	438 (77.5)	

ART, Antiretroviral Therapy, RDT, rapid diagnostic test; MP, Malaria parasites; BSM, Blood smear microscopy; n, Number examined; NS, No significant difference; *Pf*, *Plasmodium falciparum; Pv*, *Plasmodium vivax*.

*p values were calculated using Chi-square test.

### Effect of HIV and malaria co-infection on hematological parameters

The mean values of sixteen hematological parameters between HIV and malaria co-infected patients against HIV infected only patients Table 4. Although seven hematological parameters (1. hemoglobin, 2. white blood cell count, 3. red blood cell count, 4. mean cell hemoglobin, 5. mixed differential count, 6. lymphocyte differential count and 7. red cell distribution width) showed higher mean values among HIV infected without malaria compared to co-infected patients, the difference showed not statistical significant (Table 4). Conversely, co-infected patients were observed to have higher absolute neutrophil count, absolute mixed count and platelets count than those HIV infected-without malaria. These differences did not reach statistical significance (Table 4). Only six hematological parameters/indices—packed cell volume (p = 0.001), Mean cell volume (p = 0.005), mean cell hemoglobin concentration (p = 0.011), neutrophil differential count (p = 0.020), absolute lymphocyte count (p = 0.022) and platelet distribution width (p = 0.001) showed statistical significant differences between HIV only and HIV and malaria co-infected among the studied population (Table 4).

**Table 4:** Comparison of Hematological parameters Between HIV+ Patients and HIV+/Malaria+ Among Patient Attending ART Clinic at Infectious Disease Hospital Kano Between June to December 2015 (n = 761).

Hematological Parameters	HIV+ Only (n = 550)	HIV+ and Malaria+ (n = 211)	T-Test	p-values*
Hb g/dl	11.38±1.94	11.02± 2.36	1.979	0.049
PCV %	34.19 ±4.99	32.61 ± 6.39	1.583	0.001
WBC X10^9^/l	7.58±1.46	7.56±1.73	0.157	0.875
Platelet Count X10^9/l^	236±17.0	239±18.3	1.307	0.192
Red Blood CellX10^12/l^	3.91 ±0.67	3.83± 0.77	1.232	0.219
MCV fl	88.5 ±10.77	86.09 ±1.43	2.823	0.005
MCHC g/dl	33.26±2.61	33.77±2.19	-2.747	0.011
MCH Pg	29.57 ±4.82	29.17 ±4.47	1.035	0.301
Neutrophil Diff%	41.20 ±11.75	39.40 ± 12.75	-2.322	0.020
Lymphocyte Diff %	42.11 ±11.10	39.0 ±12.77	1.845	0.65
Mixed Diff %	21.88 ±0.12	21.25 ± 0.72	1.002	0.317
Abs Neutrophil X109/l	4.20 ±0.21	4.42 ±0.23	0.949	0.343
Abs Lymphocyte X109/l	3.51± 1.89	3.15 ±2.16	2.298	0.022
Abs Mixed X109/l	1.80 ±0.11	1.91 ±0.12	0.706	0.481
RDW-SD%	48.14 ±5.72	47.47 ±5.94	1.417	0.157
Platelet Distribution width	10.65 ±0.08	10.93 ±0.19	-4.871	0.001

Values are mean ± SD.

PCV-Packed cell Volume, Hb-Heamoglobin, MCV-Mean Cell Volume, MCHC-Mean Cell Heamoglobin Concentration, WBC- White Blood cell, RBC-Red blood cell, RDW-red cell distribution width, Abs-Absolute, t-Student t test, n- Number, SD-Standard deviation, P- Probability, SD-Standard Deviation.

*p values were calculated using independent student t-test.

### Comparison of CD4 count between HIV infected only and HIV and malaria co-infected

**Fig 1:**
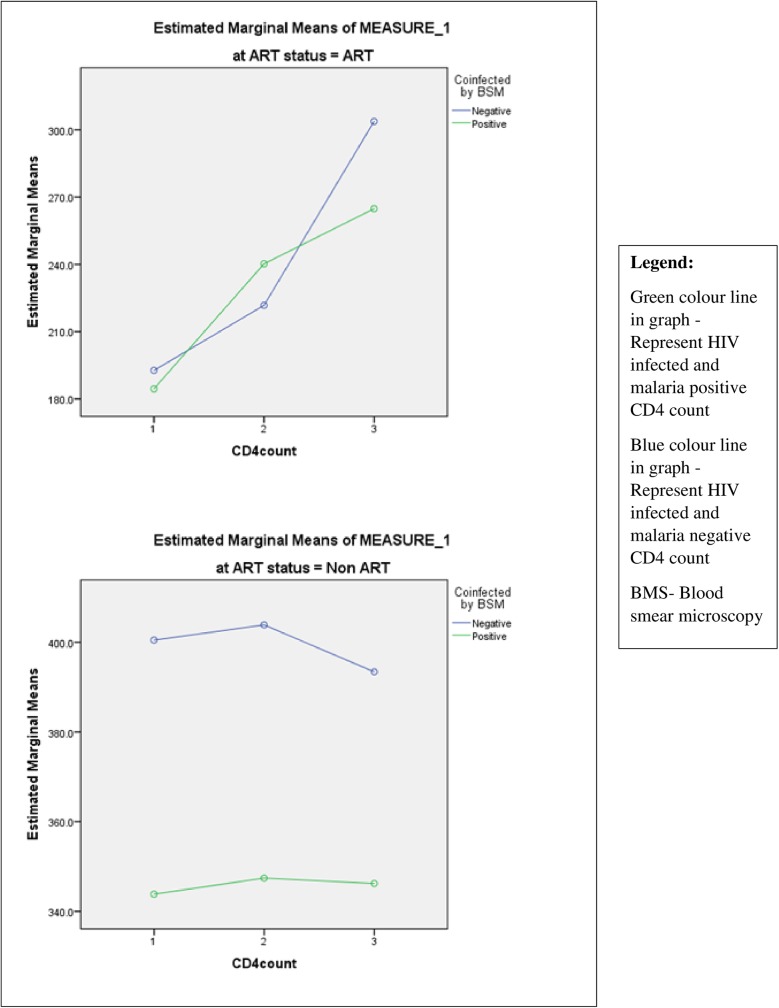
Changes in CD4+ T cell count by ART status between HIV and malaria co-infected (positive) and HIV infected only (malaria negative) patients. 1 –Baseline CD4; 2- Follow-up CD4 count; and 3- Current CD4 count among HIV patients attending ART clinic in Infectious Disease Hospital Kano from June to December 2015.

Analysis of CD4 was based on results of three CD4 count measurements carried out at baseline, follow up and most current CD4 count of patients enrolled in this study. Using a mixed design repeated measures ANOVA analysis, a significant mean difference in CD4 count over time (F = 16.347, df = 1.328, 1005.422; p<0.001) was observed among all the subjects enrolled in this study irrespective of the infection status (single or dual) (Table 5). We further examined group differences in mean CD4 count by co-infection status as well as ART status (Fig 1). We observed that overall, HIV and malaria co-infected patients had a significantly lower CD4 count (287.8 ± 12.2) compared to HIV positive without malaria (319.3 ± 8.6) (F = 4.438, df = 1, 757, p = 0.035, mean difference = -31.48). Similarly, a significantly lower mean CD4 count (234.6 ± 6.9) was observed among respondents on ART compared to those not on ART (372.5 ± 13.2) (F = 85.146, df = 1, 757, p<0.001, mean difference = -137.9). Finally, we examined group x time interaction (CD4 count*co-infection status*ART status) as the difference in change-from-baseline/first CD4 count between the four ‘treatment’ groups. Our results showed that there was no significant difference in mean CD4 count changes over time between the four groups (i.e. co-infected on ART, co-infected non-ART, HIV positive without malaria on ART, HIV without malaria non-ART) (F = 2.120, df = 1.328, 1005.422, p = 0.138) (Table 5). Changes in CD4 count in association to malaria parasite co-infection and HIV only in relation to ART status interaction presented in Fig 1.

**Table 5:** Changes in CD4+ T Cell Count Between Co-infected and Non-Co-Infected HIV Patients Attending ART Clinic at Infectious Disease Hospital Kano Between June to December 2015 (n = 761).

Outcome measure	First CD4 count	Second CD4 count	Current CD4 count	Group	Time	Group x Time
**HIV + & Malaria +**						
ART	185±13 (159–210)	240±13 (216–265)	265±17 (232–298)	0.001*	0.001*	0.138
Non-ART	344±23 (299–389)	347±22 (304–391)	346±30 (288–404)			
**HIV + only**						
ART	193±8 (178–208)	222±7 (207–236)	304±10 (284–323)			
Non-ART	400±17 (367–434)	404±16 (372–436)	393±22 (351–436)			

Values are mean ± standard error (95% Confidence Interval).

*p values obtained from mixed design repeated measures Analysis of variance.

Group x time interaction represents the ‘treatment effect’ as the difference in change-from-baseline/first CD4 count between the four ‘treatment’ groups.

## Discussion

Prevalence of HIV and malaria in this study was approximately 28% with *Plasmodium falciparum* species accounting for 99% of malaria parasites co-infection while only approximately 1% was for mixed infection of *Plasmodium falciparum* and *Plasmodium vivax*. Co-infection was significantly lower among patients who use treated bed nets as well as cotrimoxazole chemotherapy. Prevalence of malaria parasite among HIV infected was significantly higher among HIV infected non-ART compared to HIV infected on ART. The mean of six hematological indices differs between HIV only compared to HIV and malaria co-infected.

The present findings of approximately 28% HIV and malaria co-infected was similar to 24% reported in Jos [35] and 32.2% in a Kano tertiary hospital [36] but at variance with reported prevalence of 74.3% in Benin City [30] and 93.3% in Port Harcourt, [21] all within Nigeria. Such variance was also seen in a 61.7% prevalence reported in Mozambique [15]. Conversely, the prevalence of HIV and malaria co-infection observed in this study was much higher than 2.9% reported in Lagos Nigeria [3] and 18.9% in South East Nigeria [37]. Our reported prevalence was also higher than that reported in other Africa countries. For example, 15.5% [18] and 11.75% [4] in different Ghanaian studies and 10% in South Africa [20]. The prevalence of HIV and malaria co-infection observed between male and female participants in our study showed no significant difference as similarly observed by Tay and colleagues [4] and Sahle *et al*., (2017) [38]. We observed a significant lower prevalence of HIV and malaria co-infection among respondents using treated bed nets and those on cotrimoxazole. This is consistent with other research reports on the protecting ability of cotrimoxazole chemotherapy and treated bed nets in prevention of malaria among HIV infected [6][15][39]. Prevalence of malaria parasite was significantly higher among HIV infected Non-ART compared to HIV infected on ART. This was similar to a Nigerian study that reported low prevalence of asymptomatic malaria among HIV patients on ART [40]. In addition, similar patterns of lower malaria prevalence (20.5%) among HIV infected on ART compared to a 63.9% among Non-ART group in a case control study was observed in Benue, Nigeria. However, contrary to observed significant difference in our study, there was no statistically significant difference of malarial infection between patients Non-ART and those on ART (p = 0.0805) [41]. Many factors might be responsible for the variation in HIV and malaria parasite co-infection reported across states and countries. Firstly, geographical difference and other environmental factors play major role in the epidemiological diversity of these diseases as reported in Sub-Saharan Africa [5]. Secondly, variations in study design and sample size used could also be a factors given the fact that the highest prevalence of HIV and malaria co-infection (93.3%) recorded in Port Harcourt, Nigeria [21] was among only 30 HIV infected participants.

We observed that only the mean of six hematological indices showed statistically significant differences between HIV only compared to HIV and malaria co-infected among studied population (Table 4). Results of the current study was in contrast to that of Erhabor *et al (*2006) [21] which observed the incidence of pancytopenia to be significantly higher in parasitized subjects compared to non-parasitized controls. Specifically, incidence of anemia, thrombocytopenia, neutropenia and leucopenia were significantly higher in malaria parasitized subjects compared to malaria non-parasitized control [21]. Compared to hematology profiles of biological reference range among apparently healthy HIV negative adults population in Nigeria [42] only mean cell volume was similar in this study. Observed profiles were lower in packed cell volume, mean cell hemoglobin concentration, hemoglobin and red blood cells but higher in neutrophil percentage count, white blood cell, Platelets count, mean cell hemoglobin and lymphocyte percentage count. This could be partially attributed to the fact that majority of the patients (81%) were currently on ART and are being monitored regularly by ART clinicians. The prevalence of anemia in our study was moderate and similar to lower hemoglobin concentration recorded in a Ghana study as a result of HIV and malaria parasites co-infection [4]. This varied with a findings from Cameroon that reported higher prevalence of anaemia (97.1%) in HIV and malaria co-infection compared to HIV mono- infection (42.5%; p = 0.004) [18]. We also had findings similar to a Zimbabwe study which concluded that HIV-1 infection increases the incidence of *Plasmodium falciparum* parasitaemia and is associated with the development of severe malaria and mostly anemia [43]. Those hematological indices are important in monitoring malaria and HIV co-infection especially in developing countries in Sub-Saharan Africa because decrease immune response and hematological abnormalities lead to adult mortality.Therefore assessment of hematological abnormalities in malaria and HIV co-infected individual will have a remarkable benefit to prevent HIV and malaria comorbidity[44].

Part of our analysis revealed that there was no significant difference in mean CD4 count changes over time between the four groups (ie co-infected on ART, co-infected non-ART, HIV positive without malaria on ART, HIV without malaria Non-ART) (Table 5). This is probably not surprising since the effect of malaria parasitaemia is usually a short-term drop in CD4 count rather than long-term suppression. Nevertheless, it is important to note that recurrent malaria infection in non-ART patients may deplete their CD4 count to such a level that could expose them to potential opportunistic infections and warrant early commencement of ART. To this extent, it would be valuable, in our setting to adopt world health organization 2016 recommendation of test and treat strategy[45].

Our observation of prevalence of malaria and association with CD4 count was at variance with a similar longitudinal study reported among Ugandan adults that revealed no evidence of association of current CD4 with clinical malaria incidence (P = 0.56), or parasitaemia levels (p = 0.24). However, our conclusion was in consonance with the study reported that malaria occurrence did not differ by CD4 count at ART initiation, enrolment or during follow up [46]. Hence immune status of HIV infected participants who are stable on ART as measured by CD4 count was not associated with malaria incidence [46].

We observed a higher mean current CD4 count among HIV infected only compared to HIV and malaria co-infected patients Similar findings showing that the incidence of malaria was significantly higher amongst HIV patients with CD4 count <200 cells/μl compared to those with CD4 count >500 cell/μl has been reported in Malawi [17]. Furthermore, our finding is also similar to other studies in Ghana [18] and South East Nigeria [47] suggesting that HIV and malaria co-infection significantly decreases CD4 count. However, we had contrary observation to a study in Cameroon reporting that the mean CD4 count in HIV and malaria co-infection was not significantly different from that of HIV mono infection (F 0.004,p = 1.000) [14]. Such contrast too was reported in a study in Nigerian by Audu and colleagues [48]. Reason for these variations could be attributed to the choice of study participants, number of participant examined, selected participants immune status and study design. Note that in our study, majority of the patients (81%) were currently on ART and are being monitored regularly by ART clinicians. Additionally, females represents a higher percentage of our study participants and HIV infected female individuals have been reported to have significantly higher CD4 count than their male counterparts [49]. Additionally, women generally showed consistently improved immunological response to ART treatment than men [49]. Women also do present to hospital earlier than men thereby promoting better uptake of HIV treatment among women which further contribute to improve immunologic outcomes than men [50]. However, evidence from a Zambian study seems to have provided useful suggestion that interpretation of absolute CD4 count in HIV infected might be biased during or just after a clinical malaria episode. Thus before taking any decision on the management of HIV positive individuals, in malaria-endemic areas, their malaria status should be evaluated [51].

### Strength and limitation of the study

We evaluated a total of sixteen hematological profiles and three CD4 count longitudinal measurements at baseline, follow-up and current. Thus we were able to comprehensively study patients’ CD4 count performance over a period of time including relationship with the hematological profiles. In addition, we had a relative large sample size of studied population.

A major limitation was our inability to determine the viral load of the participants to relate it with hematological parameters due to logistic reasons. Secondly, the cross sectional design nature of the study. Thirdly, the hematology analyzer Sysmex XP-300 available at the facility for routine hematology investigations was limited because it is a three-part differential unlike the five-part differential count. Hence both percentage differential and absolute count of monocytes, eosinophils and basophil count were reported as mixed population which could have provided additional useful hematological indices.

Further comprehensive studies focusing on HIV and malaria (major diseases of public health impact) interaction and implication on viral load and hematological profiles is desirable in Sub Sahara Africa.

## Conclusion

HIV and malaria co-infection burden is high among studied population with no statistical significant difference in age or gender. HIV and malaria co-infection was significantly lower among patients who use treated bed nets as well as cotrimoxazole chemotherapy and ART. Immune recovery of HIV positive patients (often monitored by change in CD4 count) may be affected by malaria co-infection (especially recurrent malaria infection) and ART status. Six blood count indices (mean cell volume, packed cell volume, absolute lymphocyte count, neutrophils percentage count, platelets distribution width and mean cell hemoglobin concentration) were significantly different between HIV positive only compared to HIV and malaria co-infected.

Overall, to achieve better management of all HIV patients in this setting, malaria prevention through cotrimoxazole chemotherapy, and use of treated bed nets are useful strategy. Diagnosing malaria, prompt antiretroviral therapy, monitoring CD4 count and some hematology indices on regular basis is critical.
